# Monitoring and Prediction of Siberian Silk Moth *Dendrolimus sibiricus* Tschetv. (Lepidoptera: Lasiocampidae) Outbreaks Using Remote Sensing Techniques

**DOI:** 10.3390/insects14120955

**Published:** 2023-12-15

**Authors:** Vladislav Soukhovolsky, Anton Kovalev, Andrey A. Goroshko, Yulia Ivanova, Olga Tarasova

**Affiliations:** 1 V.N. Sukachev Institute of Forest SB RAS, 660036 Krasnoyarsk, Russia; 2 Krasnoyarsk Scientific Center SB RAS, 660036 Krasnoyarsk, Russia; sunhi.prime@gmail.com; 3 Scientific Laboratory of Forest Health, Reshetnev Siberian State University of Science and Technology, 660037 Krasnoyarsk, Russia; utrom3@gmail.com; 4 Institute of Biophysics SB RAS, 660036 Krasnoyarsk, Russia; lulja@yandex.ru; 5 Department of Ecology and Nature Management, Siberian Federal University, 660041 Krasnoyarsk, Russia; olvitarasova2010@yandex.ru; 6 Institute of Systematics and Ecology of Animals, Siberian Branch of Russian Academy of Sciences SB RAS, 630091 Novosibirsk, Russia

**Keywords:** forest stand, forest insect, population outbreak, monitoring, forecast, remote sensing data

## Abstract

**Simple Summary:**

Insects are a significant cause of harm and mortality to forest areas. In particular, the forests of Siberia, covering over 2.4 million square kilometers with a low or non-existent pest insect population, are at high risk. Under such conditions, observing insects from the ground is nearly impossible. Consequently, remote sensing techniques are the sole feasible option. However, many remote indicators only change after significant damage occurs to the stands of trees, making it too late to implement protective measures. The objective of this study was to construct distance indicators that could identify areas where forest stands have lost resistance to attacks by such a dangerous pest of Siberian forests as the Siberian silk moth *Dendrolimus sibiricus* Tschetv. To create indicators, it has been suggested that estimations rely on the reaction of stands to weather conditions during the season, instead of absolute values. Such remote indicators were constructible, and these indicators in the areas of future outbreaks had changed already two or three years before the outbreak. Remote sensing data allow the development of complex indicators of forest stands’ insect resistance anywhere in the world, thus making it possible to monitor large areas to identify risk of pest outbreaks.

**Abstract:**

The feasibility of risk assessment of a Siberian silk moth (*Dendrolimus sibiricus* Tschetv.) outbreak was analyzed by means of landscape and weather characteristics and tree condition parameters. Difficulties in detecting forest pest outbreaks (especially in Siberian conditions) are associated with the inability to conduct regular ground surveillance in taiga territories, which generally occupy more than 2 million km^2^. Our analysis of characteristics of Siberian silk moth outbreak zones under mountainous taiga conditions showed that it is possible to distinguish an altitudinal belt between 400 and 800 m above sea level where an outbreak develops and trees are damaged. It was found that to assess the resistance of forest stands to pest attacks, researchers can employ new parameters: namely, characteristics of a response of remote sensing variables to changes in land surface temperature. Using these parameters, it is possible to identify in advance (2–3 years before an outbreak) forest stands that are not resistant to the pest. Thus, field studies in difficult-to-access taiga forests are not needed to determine these parameters, and hence the task of monitoring outbreaks of forest insects is simplified substantially.

## 1. Introduction

Forest insects are the second (after forest fires) most significant factor in terms of damage to (and death of) Siberian forests. It is possible to protect a forest from attacks by many species of forest insects, but timely pest control requires monitoring and predicting the state of pest populations and the resistance of forests to insect attacks. In many cases, however, it is very difficult to directly analyze the status of insect populations during outbreak development. In particular, for taiga forests of Siberia with a total area of ~2.7 million km^2^, it is absolutely impossible to keep records of abundance of the main forest pest—the Siberian silk moth *Dendrolimus sibiricus* Tschetv. (Lepidoptera: Lasiocampidae)—throughout the entire territory of the taiga.

Effective mitigation of damage during pest insect outbreaks requires reliable models that predict areas where an outbreak may occur and where the risk of tree mortality is high. Modeling insect damage requires taking into account forest stand structure, environmental factors, and differences in site topography [1]. Most studies on the modeling of forest insect outbreak risk are based on variables related to forest stand characteristics and to insect abundance [1,2,3,4,5].

In many cases, however, it is challenging to directly analyze the status of pest insect populations in the course of outbreak development. Recent research makes it clear that pest outbreaks are more complicated than previously thought. Most often, insect pest outbreaks are caused by factors operating at multiple levels, i.e., host abundance at the forest stand level, landscape characteristics, and climatic factors at the geographic-region level [6,7,8]. Typically, the growth of pest populations is described at the stand level using population models or system dynamics [9,10,11]. This modeling, however, does not explain how local events translate into large-scale outbreak dynamics.

Successful pest monitoring necessitates a comprehensive approach to insect outbreaks that describes the spread of pests. Mathematical models of multiscale dynamics of outbreaks are needed and may lead to improved outbreak forecasting and to the development of effective preventive actions mitigating outbreak-caused damage. At present, available high-resolution spatial data on pest outbreaks are used to achieve this goal. Remote sensing can be an effective tool for timely large-scale monitoring of pest infestations at their different phases [12,13]. For example, in North America, remote sensing has been utilized to detect tree mortality caused by the mountain pine beetle [12,13,14,15,16,17,18,19,20].

On the other hand, remote sensing techniques have serious limitations related to the spatial resolution of the available data. Insects are orders of magnitude smaller than the spatial resolution (pixel size) of many remote sensing devices, whereas short life cycles of many pest species mean that phenomena important for their monitoring and detection can be easily overlooked at low temporal resolution [6]. Consequently, both insects themselves and small-scale features of their habitats are hard to detect remotely [12,21]. Despite these limitations, applications of remote sensing have a long history in entomological research [21,22]. In particular, much effort has been devoted to the detection and monitoring of insect pests in agriculture and forestry [6,23,24,25].

The ability to remotely sense habitat structure also enables research on insect–microclimate interactions. Climate plays a fundamental role in mechanisms governing the physiology and ecology of organisms by strongly affecting the abundance, phenology, and geographic ranges of species [26]. Nonetheless, until recently, there has been a large discrepancy between the scale at which climate variables are traditionally measured and the scale at which they are sensed by insects [27]. Climatic factors are influenced by topography and by the vegetation type, which cause small-scale microclimatic changes that are maintained by low wind speeds [28]. These microclimates characterize the thermal environment of most insects but are often highly heterogeneous and differ markedly from regional climates’ average parameters measured at standard weather stations [29]. The inability to capture these small-scale climate alterations over geographically large areas is a major limitation of most studies on insect climate to date [27,29].

Insect feeding activity causes biomass loss and a plant stress response, which can be detected by means of their spectral reflectance. Therefore, such effects as defoliation and plant stress symptoms caused by insects are often easy to detect by remote sensing, and these parameters have long been used for the indirect detection of insects [22]. Thus, remote sensing allows for automating the monitoring of insect outbreaks over large areas, and such surveillance by means of conventional field surveys alone would otherwise be prohibitively expensive and time-consuming [25]. For this purpose, plant indices derived from multispectral satellite data are widely employed, especially in the context of the monitoring of disturbances related to forest insects [6]. The main metric among them is the normalized difference vegetation index (NDVI), which, as an indicator of photosynthetic activity, is sensitive to defoliation and plant death. In particular, NDVI data obtained by satellites have been used to map spatiotemporal patterns of forest defoliation caused by gypsy moths [30,31] and by sawfly larvae [32], as well as the tree mortality caused by bark beetles [33,34]. High-resolution temporal data are generally important for the detection of defoliating pests [35]. Historically, most defoliator monitoring studies have had to rely on coarse spatial data, such as measurements by the Moderate Resolution Imaging Spectroradiometer (MODIS) Aqua/Terra satellite system, to obtain desired temporal resolution [12,36].

For the fragmented forest landscapes of Central Europe, MODIS-type data are less suitable due to their low spatial resolution. For this reason, Landsat data have been widely used in the Central European region [37,38,39]. Spatiotemporal merging of MODIS and RapidEye data having high spatial resolution [20] as well as combinations of Landsat and SPOT data [40] have been investigated in terms of determining tree mortality caused by the bark beetle in the Bavarian Forest National Park. Lately, studies have been addressing pest detection based on Sentinel-2 data, as well as tree mortality mapping [41,42,43]. Nevertheless, most of such research has been focused on mapping the stages of an insect attack on trees, tree mortality, or a forest disturbance in general, but such methods are unsuitable for the early detection of infested trees (before the onset of symptoms visible to the human eye) and therefore cannot help to effectively plan such management measures as emergency logging [44].

NDVI-based indices have gained popularity owing to their good performance in earlier studies on the mapping of insect attack stages [15,20,45]. There is evidence that at an early stage of damage to Norway spruce [46] and to Engelmann spruce [47], there are indeed spectral differences at the leaf level between healthy and damaged trees. Pronounced spectral differences were found in the red region of the visible spectrum [46]. The far-infrared region also proved to be informative for assessing the condition of trees [47]. Nevertheless, distinguishing healthy and damaged trees at the forest canopy level is a challenge because of the structural complexity of forest canopies and owing to the scaling of a leaf-level signal to the level of entire forest stands [48].

Most previous studies of tree–insect interactions using satellite-based methods [12,15,16,18,19,20,49] and unmanned aerial vehicles [50,51,52] have focused on analyzing changes in spectral characteristics of stands in the early stages of insect damage to trees. Using data from seasonal dynamics of vegetation indices is preferable to individual observations for early detection of damage with a probability of *p* = 0.75 of separating healthy and damaged stands [49]. However, these methods cannot identify areas that will be attacked by insects in the near future, as they are time-lagged. Assessments of forest health using such methods cannot be used to identify areas that will be attacked by insects in the near future. The use of remote sensing methods in this context solely measures damage extent, which is significant for forest economics and inventory purposes, but not for forest protection. Unlike the mentioned approaches, the aim of this study is to locate the possible site of the outbreak prior to insect attacks on the forest.

Thus, to predict the development of insect outbreaks within a small territory at certain points in time, it is difficult or impossible (especially on giant taiga territories) to use data on insect population density; to solve the problems of the monitoring and prediction of outbreaks, it is necessary to take advantage of remote sensing data. At this point, a question arises: which of the numerous remote indicators will be most useful for solving the problems of monitoring and forecasting pest outbreaks?

Because on the vast territories of Siberian forests, it is physically impossible to monitor sizes of insect populations before an outbreak begins; it is instead necessary to identify the necessary and sufficient conditions whose fulfillment within a specific territory leads to the development of outbreaks. For retrospective analysis, however (only this kind of analysis is really possible because it is unknown where an outbreak may occur and therefore it is impossible to assess many characteristics of an ecosystem before the outbreak), only a limited number of parameters can be used. Such parameters include weather and landscape characteristics of the territory where the outbreak occurred and remote sensing data on the territory before the outbreak.

The present work is based on data from a retrospective analysis of an outbreak of *D. sibiricus* in mountain forests in the south of Central Siberia in the years 2019–2020. By means of these data, necessary and sufficient conditions for the development of a massive outbreak were assessed, and the feasibility of predicting an outbreak zone was examined. For the analysis, we used remote sensing data on seasonal dynamics of photosynthetic index, NDVI, and land surface temperature (LST), as determined by Aqua/Terra satellites in the years preceding the onset of the active phase of the Siberian silk moth outbreak. For the years of the outbreak, we analyzed weather, landscape characteristics, and species composition of the territories in which the outbreak occurred.

## 2. Materials and Methods

The Siberian silk moth is a species endemic to forests of Siberia, of the Russian Far East, and of Northern China and is characterized by good flight abilities at the adult stage and by regular outbreaks across large territories, during which damage to and death of forest stands can take place [53,54,55,56,57,58,59,60,61,62,63,64,65,66,67,68]. Investigation into population dynamics of the Siberian silk moth in Siberia has been active for more than a hundred years, but in the literature, there is a description of only two fairly long time series of population dynamics. The reason for this is that entomological research on this species is conducted in difficult-to-access taiga forests with an extremely low density of a human population where there are no roads. In this situation, it is almost impossible to estimate population density of the Siberian silk moth at the stage of the stable sparse state of its populations: the average abundance of individuals can be less than 0.001 caterpillars per tree. Of course, at the peak phase of an outbreak, its population density can rise to approximately 10,000 individuals per tree; that is, the range of this pest’s population density can reach seven orders of magnitude [57]. Nevertheless, if a researcher has to predict an outbreak, it is almost impossible to use data on the insects themselves for the analysis. Outbreaks practically begin only when the insects damage forest stands so severely that this change becomes visible even via satellite observations. Furthermore, only in the context of elevated population density does it become possible to assess the influence of parasites on the dynamics of the pest population. Nonetheless, even if an area of an initial outbreak is discovered, it is very often extremely hard to reach it, and the trip to the site is realistically only possible on a helicopter.

In studies on the impact of the Siberian silk moth on forest stands via remote sensing methods, investigators usually analyze the extent of damage to forest stands [63,67]. Unfortunately, such postoutbreak studies do not permit early assessment of the risk of outbreaks and allow only for post factum quantification of the impact that has already occurred. These data may be interesting in terms of forest damage statistics but do not help to identify the environmental factors that led to the outbreak.

The objective of the present project was to develop remote methods that will make it possible to quickly evaluate changes in the state of forest stands and in their resistance to pest attacks as well as to assess the risk of outbreaks beforehand, so that it becomes possible to manage forests’ conditions and to prepare the measures for protecting forests from pest attacks in advance.

**Figure 1:**
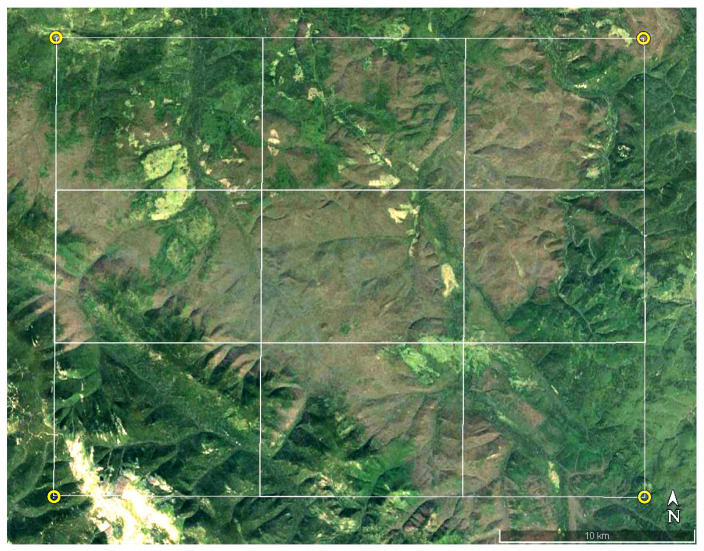
Satellite image (Google Maps, https://www.google.com/maps, accessed on 15 July 2023) of the study area within the boundaries (54°45′–55°05′ N, 95°20′–99°10′ E) after the 2019–2020 Siberian silk moth *D. sibiricus* outbreak.

In this study, we analyzed an outbreak of the Siberian silk moth in the Irbeyskiy District of Krasnoyarsk region (years 2019–2020) in mixed fir–cedar forest stands in the Sayan Mountains (southern Central Siberia, coordinates: 54°45′–55°05′ N, 95°20′–99°10′ E). The territory is dominated by dark coniferous forest stands: Siberian pine (*Pinus sibirica*), fir (*Abies sibirica*), and spruce (*Picea obovata*). Deciduous tree species (*Populus tremula* and *Betula* spp.) occupy ~15% of the territory. Visible damage to needles in the forest stands was recorded in 2019, and by 2021, the growth of the damage stopped, and the outbreak subsided (Figure 1).

The approach to the evaluation of the state of forest stands that was used in this work represents a transition from assessments of the state of environmental objects (e.g., the assessment of the NDVI) to the evaluation of a response of these forest objects to external factors. To implement this approach, it is necessary that exogenous dosed disturbances be applied to forest stands throughout the taiga. The only type of disturbance that can be used for such comprehensive monitoring is weather changes.

For this purpose, on the territory damaged by the Siberian silk moth and in nearby undamaged forest stands (a control), seasonal time series of NDVI and LST data from Aqua/Terra satellites were acquired at nodes of a square grid with a characteristic size of 250 m that was superimposed on the entire zone. Thus, we obtained a rectangular array of 226 × 102 points with time dynamics of NDVI and LST data for each point.

Additionally, altitudes (in meters above sea level) for all analyzed nodes of the superimposed lattice were taken into account. The analysis was carried out using a NASA database, Sentinel-2 satellites [69,70], and surveys involving unmanned aerial vehicles.

**Figure 2:**
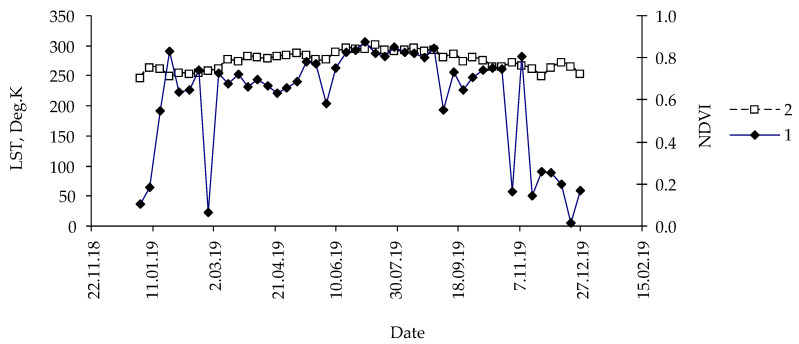
Typical time dependence of indices: 1. NDVI, 2. LST. Pixel coordinates: 95.59687 and 54.87187; fir forest, control, year: 2014.

Traditionally, remote sensing data are presented as a graph of time versus a sensing variable (Figure 2).

In the course of a year, however, the NDVI decreases depending on the extent of removal of tree leaves by insects, and until the onset of damage, the NDVI is stationary in the middle of a year. We needed to identify the changes in remote indicators that manifest themselves before an outbreak as necessary conditions for the loss of tree resistance to pests.

In this regard, for years *j* (*j* = 2005–2021), relations between intra-year series of LST(*i*,*j*) and NDVI(*i*,*j*) (*i* = 1–48) were analyzed. Quantity *T*(*s*,*i*) (average temperature of the surface of a pixel: 250 × 250 m) for three adjacent measurements (*i* − 2), (*i* − 1) and *i* served as a characteristic of an external temperature field during year *j*. As parameter of NDVI(*i*), a quantity was introduced that we named the order parameter squared: *q*^2^(*i*) = (1 − NDVI(*i*))^2^. Because 0 ≤ NDVI ≤ 1, *q*^2^ should also change from 0 to 1.0.

When the NDVI reaches maximum values during a year, close to 1.0, *q*^2^ approaches 0. In winter, when the crowns are completely or partially covered with snow and chlorophyll is not detectable remotely, NDVI approaches 0 and *q*^2^ approaches 1.0.

Trajectories of *q*^2^ dynamics differ between spring and autumn. At the same LST values, *q*^2^ is greater in spring than in autumn, and the curve of the intra-year relation between accumulated surface temperature *T_s_* and NDVI forms a kind of hysteresis loop.

Our analysis of observational data showed that the intra-year dynamics of the NDVI can manifest either thermally soft properties, when the width of the hysteresis loop is small, or thermally rigid properties, when the width of the hysteresis loop is sufficiently large.

Derivative d(ln *q*^2^)/d*T_s_* = *B* in Equation (1) characterizes the susceptibility of changes in NDVI to changes in *T_s_*.

To describe the hysteresis curve during an assessment of the dependence of ln *q*^2^ on *T_s_*, the curve’s branches must be described by separate equations. Nonetheless, remote sensing data are characterized by a high level of noise, which leads to a distortion of the hysteresis curve and does not allow a researcher to unambiguously identify objects with thermally soft properties or thermally rigid properties or to derive regression equations for them in phases of intra-year dynamics.

**Figure 3:**
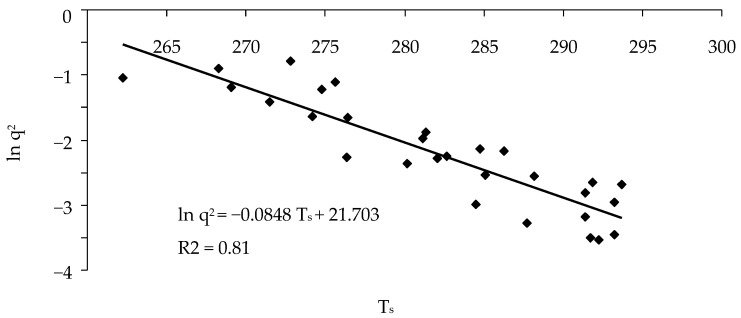
The relation between the logarithm of the order parameter squared *q*^2^ and *T*_s_ during a year.

In this case, the intra-year dependence of ln *q*^2^ on *T_s_* is described by one linear regression equation (Figure 3):

(1) $$\ln q^{2}=A-BT_{s}$$

Coefficient of determination *R*^2^ will depend on the variance of *q*^2^ values and on the width of the hysteresis loop. In this context, the wider the loop of the hysteresis curve is, the lower the coefficient of determination *R*^2^ of the regression equation. Quantity *A*/*B* = *T*_0_ characterizes the average value of LST at which ln *q*^2^ will reach 1.0 (that is, a minimum NDVI will be reached).

Thus, each pixel of remote sensing data in the studied area can be described by the three numbers {*A*, *B*, *R*^2^} and by quantity *T*_0_ that derives from them. Using remote sensing data collapsed in this way, one can proceed to search for relations between characteristics of remote sensing and the likelihood of outbreaks in an area sometime after changes in LST and in the susceptibility of the NDVI to changes in LST.

Based on remote sensing data within the study area, altitudes (in meters above sea level) for all grid nodes were estimated, and data on intra-year dynamics of LST were obtained for 5 years before the onset of visible damage.

## 3. Results

**Figure 4:**
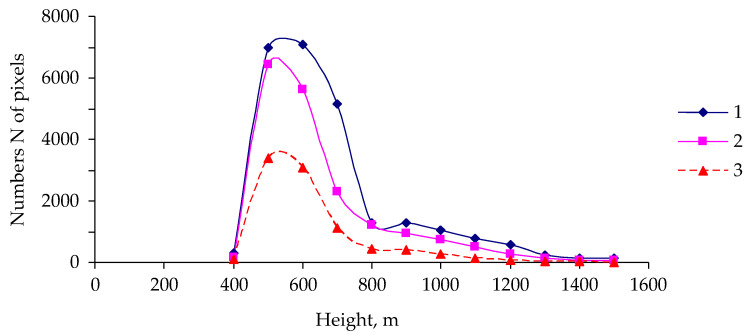
Characteristics of pixels in the surveyed area. 1: The total number of pixels at altitude H; 2: the number of pixels at altitude H with forest stands of predominantly coniferous species; 3: the number of pixels at altitude H with predominantly insect-damaged forest stands.

At the first stage of the analysis, we examined the relation between the risk of an outbreak and characteristics of the distribution of the pest’s damage to trees depending on the altitude of a forest stand. By means of remote sensing data, altitudinal classification of territories was performed in the outbreak zone (Figure 4).

As one can see, most of the surveyed territory is located at altitudes of 400 to 800 m above sea level and is covered mainly by coniferous forest stands.

Figure 4 shows the number of pixels with predominantly coniferous forest stands at various altitudes in the outbreak zone and the number of pixels with damaged forest stands. To calculate these parameters from remote sensing data, altitudes were determined for each pixel in a grid node; in the period before the outbreak (in the year 2018), by means of autumn phenological data, pixels having NDVI values > 0.60 were identified, which characterize trees from which needles had not fallen by this time point in intra-year dynamics.

As presented in Figure 4, most of the studied area is covered with forest stands of predominantly coniferous species.

To estimate the number of pixels with damaged stands, pixels with NDVI values < 0.60 in the fall of 2021 were selected from the pixels with predominantly coniferous trees.

**Figure 5:**
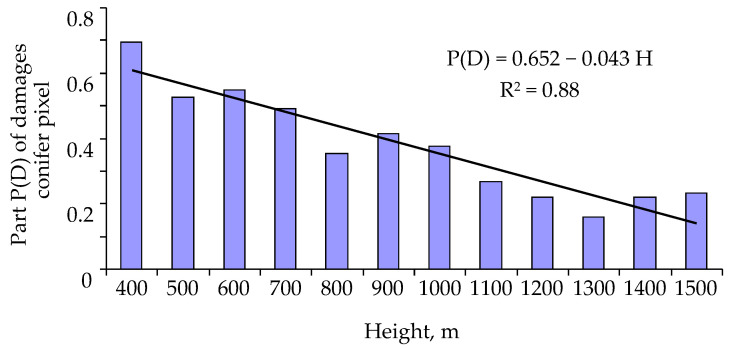
The proportion of pixels with pest damage among pixels with predominantly coniferous forest stands.

Among pixels with predominantly coniferous forest stands, the proportion of pixels with pest damage diminishes with altitude (Figure 5).

From Figure 5, it follows that with increasing altitude, the susceptibility of trees to a pest attack decreases linearly and that $\frac{\partial P(H)}{\partial H}=-0.043$ . It should be noted that for mountain forest stands of Siberia, the average air temperature decreases by 5 °C with an increase in altitude by 1 km [71].

**Figure 6:**
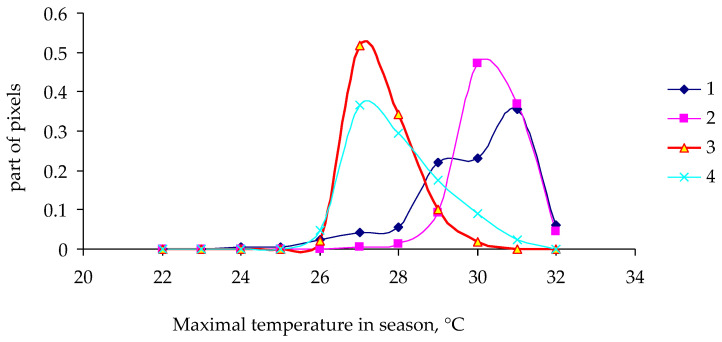
Functions of the density of distribution of pixels by maximal temperature during a year in not affected (NA) and insect-affected (A) stands at altitudes of 400 to 800 m. 1: NA, 2014; 2: A, 2014; 3: NA, 2017; 4: A, 2017.

**Figure 7:**
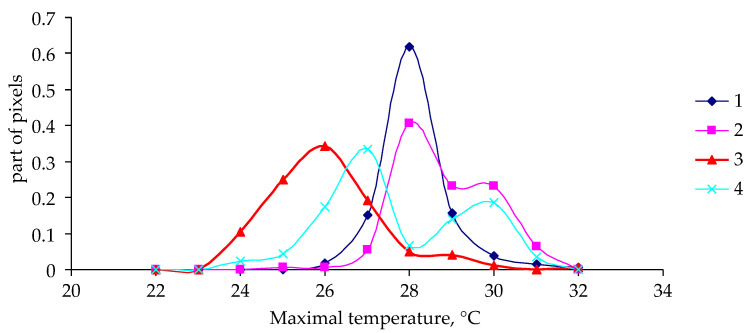
Functions of the density of distribution of pixels by maximal temperature in a pixel during a year in not affected (NA) and insect-affected (A) stands at altitudes of 400 to 800 m. 1: NA, 2018; 2: A, 2018; 3: NA, 2019; 4: A, 2019.

After examination of the data on the altitudinal classification of outbreak sites, a question arises about a possible influence of weather conditions on the development of the outbreak. Indeed, as the altitude of forest stands increases, the weather becomes colder (less tolerable for insects), and this can explain the decrease in the proportion of pixels with damage. Nonetheless, it is more important to evaluate the weather change as a necessary (or perhaps sufficient) condition for an outbreak. In Siberia, weather conditions are synchronized over a fairly large area. For instance, in the Lower Yenisei zone, the length of correlating weather conditions reaches 250–300 km; that is, weather is synchronized over an area of approximately 50 thousand km^2^, whereas the zone of an outbreak in such an area is much smaller. For the 2014–2021 period, the highest temperatures (in °C) during a year were determined for pixels with damaged coniferous forest stands at altitudes of 400 to 800 m above sea level. Figure 6 and Figure 7 show functions of the density of distribution of pixels by maximal temperature in a pixel during a year.

As one can see in Figure 6 and Figure 7, the shape of the distribution density function (dependence on maximal temperature in a pixel) differs little between damaged and undamaged forest stands in 2014 and in 2017. For the year 2018, the distribution density function for undamaged stands is slightly shifted to the region of lower temperatures, whereas for 2019 (the year of onset of visible damage to tree crowns), the functions of the density of distribution of pixels by maximal temperature were not much different between damaged and undamaged forest stands.

**Figure 8:**
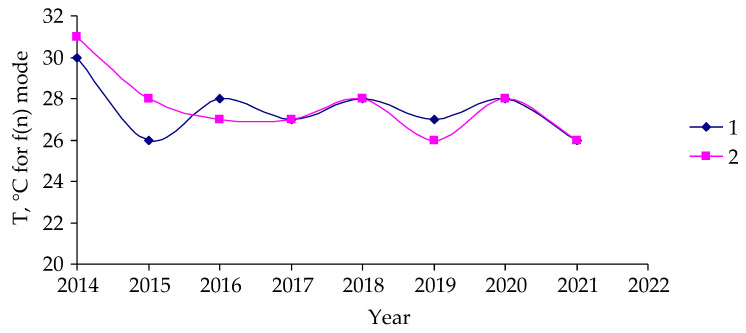
Temporal changes in the maximum temperature pixel’s temperature distribution function were analyzed for the period of 2014–2021. 1: Data from attacked forest stands; 2: data from unattacked stands.

No substantial differences between damaged and undamaged forest stands were found in temperatures corresponding to the maximum of these functions (Figure 8).

Therefore, for the outbreak under study, there is no reason to believe that it was caused by temperature changes in the area that would become an outbreak zone.

Naturally, an outbreak can start if there is a zone in which certain nonzero (even small) density of pest population persists in combination with the presence of trees that are not resistant to the attack by pests in some territory section having favorable landscape and weather conditions. Of course, under the conditions of the Siberian taiga, it is not possible to assess the density of a pest population before an outbreak, but one can try to evaluate the condition of forest stands in the outbreak zone.

For this purpose, characteristics of growth processes in trees damaged by the insects were examined, and the same was observed for trees in undamaged control forest stands. The forest stands in the outbreak zone and the undamaged stands were similar in inventory characteristics: tree height, diameter, stand density, and age (~80–100 years).

Because it is possible that the onset of an outbreak may be associated with a change in the conditions of trees in the territory in question, it is reasonable to ask, is it possible to assess the conditions of forest stands using remote sensing data? For this purpose, we analyzed the dynamics of the aforementioned parameters, *T*_0_ and *R*^2^, for all pixels in the 2010–2020 period (that is, before the outbreak, during the outbreak, and after the end of the outbreak) in the investigated area.

**Figure 9:**
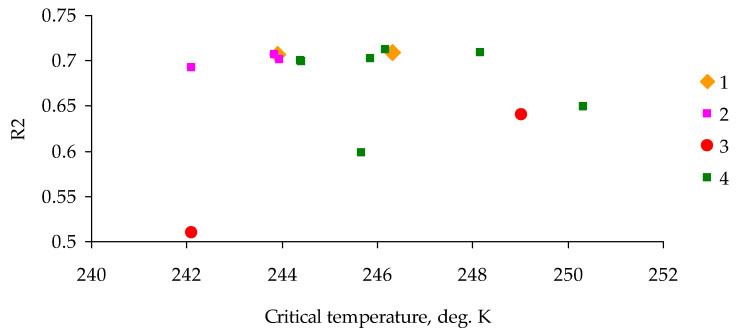
Differences in intra-year remote sensing integral parameters *T*_0_ and *R*^2^ between the outbreak zone and control area (Irbey, 8,480,560 records total) for years 2014–2020. 1: Future outbreak zone, 2014–2015; 2: future outbreak zone, 2016–2018; 3: outbreak zone, 2019–2020; 4: control zone, 2014–2020.

Figure 9 depicts intra-year average values of integral remote sensing parameters *T*_0_ and *R*^2^ in the outbreak zone and control area (8,480,560 records total) for 2014–2020.

Before the outbreak, the coefficient of determination *R*^2^ was close to 0.7 both in pixels of the outbreak zone and in pixels of the control area, where *T_0_* (in degrees Kelvin) was no less than the critical value of *T_c_* = 244 K. For zones of the already developed outbreak of the pest, the coefficient of determination *R*^2^ declined to 0.6, and the value of *T*_0_ became less than 244 K.

Next, *T*_s_ values were transformed to binary scale *U*:

(2) $$U=\left\{\begin{array}{l} 0,\;T_{0}\leq T_{c},\;\mathrm{red}\;\mathrm{dots}\;\mathrm{in}\;\mathrm{the}\;\mathrm{figures}\\ 1,\;T_{0}>T_{c},\;\mathrm{blue}\;\mathrm{dots}\;\mathrm{in}\;\mathrm{the}\;\mathrm{figures} \end{array}\right.$$

**Figure 10:**
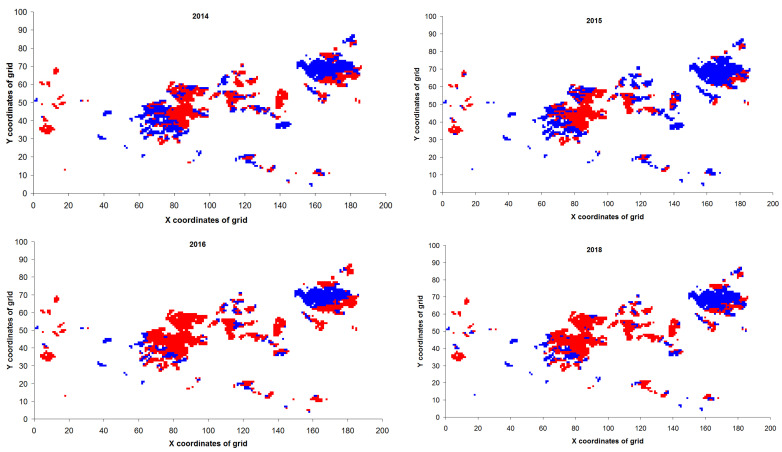
The spatial distribution of *U* values for the outbreak zone in the year 2019 and at a different years before the outbreak. Red dots: *U* = 0; Blue dots: *U* = 1.

Based on remote measurements and calculations of parameters via Equation (2), *U* values were computed for each pixel on the territory that would become an outbreak zone (Figure 10).

Proportions of pixels with *U* = 0 on the territory that would become the outbreak zone in 2019 are presented in Table 1.

Therefore, already by the year 2016 (that is, 3 years before the onset of damage), contours of the future outbreak zone became visible, and the zone of the future outbreak can be identified in advance by means of parameters in the equation describing the relation between the logarithm of the order parameter squared and accumulated LST. Because the coefficient of determination *R*^2^ average values for data in areas that would become outbreak zones were close to 0.70, this means that the hysteresis curves of remote sensing data in these risky areas can be described as thermally soft. By contrast, during the development of the outbreak, when plant damage occurred, remote sensing data changed in this zone (*R*^2^ ≈ 0.50–0.65), and the changes in these data proceeded in a thermally rigid mode. The small proportion of pixels with *U* = 0 in 2020 is likely due to the appearance of herbaceous vegetation in the satellite data after insect destruction of conifers.

## 4. Discussion

The analyzed characteristics of outbreak zones of the Siberian silk moth cannot be considered sufficient conditions for an outbreak to occur. In the absence of reliable data on the current dynamics of this pest’s abundance, the analyzed parameters (landscape and weather conditions, characteristics of regulatory processes during the growth of woody plants, and characteristics of a response of the remote sensing parameter NDVI to changes in surface temperature (LST)) can be regarded as necessary but insufficient conditions for outbreak initiation.

Forecasting the stability of forest stands and identifying areas where forest insect outbreaks are possible are extremely important for solving forest protection problems. For the vast territory of the Siberian taiga, only remote sensing methods can be effectively used to solve the tasks of forest condition monitoring and assess the resistance of stands to insect attacks. When carrying out remote sensing measurements, current characteristics of stands condition, such as the Normalized Difference Vegetation Index (NDVI), Normalized Differential Water body Index (NDWI), Ratio Vegetation Index (RVI), Differential Vegetation Index (DVI), Renormalized Difference Vegetation Index (RDVI), Enhanced Vegetation Index (EVI), Transformed Vegetation Index (TVI), Red Vegetation Index (RVI), Normalized Difference Index (NDI), and Near-infrared Divided by Green (NIRG), are usually used [72,73,74,75,76,77,78,79,80].

However, the indices that are used to evaluate the effect of the Siberian silk moth on taiga forest areas only describe the current conditions of the stands. By using these indices, it is possible to determine the level of damage, but not the resistance of the stands to possible future impacts [68].

Due to the inefficiency of current remote sensing indices in evaluating stand stability and attractiveness to forest insects, a new approach to the use of remote sensing data is needed. This study proposes an approach that is not based on the current values of a given index *I*, but on indicators of the susceptibility $\chi =\frac{\partial I}{\partial F}$ of this index to certain external factors *F*.

Since the areas of potential future outbreaks are unknown, it is necessary to estimate the value of *χ* for all stands in the study area, and therefore, it is necessary to know the intensity of this effect at any point in the forest area. It appears that the only factor affecting any stand that can be quantified by remote sensing is the land surface temperature (LST).

In addition to the current susceptibility index, it is necessary to consider the inertia of the temperature effect on the current value of the selected remote index. With this in mind, NDVI was selected as the current state index, and the average LST value over a period of time was chosen as the external factor integral. Since the level of NDVI response inertia to temperature change is unknown, different possible inertia indices *τ* ranging from 2 to 5 days were evaluated. Comparing the inertial susceptibility values of *χI* at varying τ demonstrated that the most optimal susceptibility value is *χ*(*τ =* 3). Therefore, it is necessary to consider the effect of the average temperature on NDVI over the past three days. Additionally, it has been proposed to estimate susceptibility not only for the current date but for the entire season. By doing so, the stand’s resistance to insect attacks can be evaluated by the variability in the current susceptibility *χ*(3) during the duration of the season. As a means to assess a forest stand’s resistance to insect infestation, we suggest using indicators based on regression coefficients of the relationship between the integral accumulated indices of the LST index and the susceptibility of the index *χ*(3) to changes in the LST.

Thus, the stand’s resistance to pest insect attacks was calculated through multiple stages. Initially, the current NDVI and LST values during the season were estimated, followed by a transition from the series of NDVI and LST values to the series of the first differences of these indicators to eliminate seasonal trends. For each day on which remote sensing measurements were taken, we estimated the average LST characteristics for the previous three days. From there, we calculated a regression equation to determine the relationship between the logarithm of the square of the inverse current NDVI value and the integral LST value. The resulting coefficient provides a single coefficient to describe the resistance of stands to insect attack. The purpose of transforming the NDVI index was to create linear relationships between the transformed NDVI index and the integral LST index. The proposed model was then tested with retrospective data analyzing forest damage during the Siberian silk moth outbreak in the territory, concluding the last stage of our research. The whole procedure was performed for each elementary cell (250 × 250 m) of the study area.

As shown in the paper, the value of the index *χ*(3) characterizes the area of a future outbreak about 2 years before it starts. Based on the estimated low level of insect damage in the stands using the NDVI index, it can be assumed that the pest population was low two years before the outbreak, and ground surveys would not have detected an increase in the pest population. Since Siberian silk moth outbreaks are possible in mountainous conditions within the altitude range of 400 to 800 m A.S.L., locating forests at these altitudes becomes a necessary condition for their outbreak. To monitor the risk of Siberian silk moth outbreaks in mountainous conditions, it is crucial to measure the proposed distance indicators at the altitude that is most optimal for the development of an outbreak of Siberian silk moth.

Similarly to the explanation of the link between growth processes in trees and their resistance to an insect attack, one can also explain the connection between the resistance of trees and the response of remote sensing variables to changes in temperature parameters. The slower the response of the NDVI to changes in LST observed in stands that are later attacked by insects, the stronger the inertia of woody-plant characteristics is and the slower the rate of response is to an insect impact. The availability of remote sensing data for all taiga territories opens up an opportunity to use them for the rapid monitoring of forest stands’ conditions and of their resistance to pests.

## 5. Conclusions

It is impossible to conduct regular surveys of abundance dynamics of insect pests throughout the gigantic territory of taiga forests. Accordingly, the feasibility of risk assessment of an outbreak—by means of landscape and weather characteristics and tree condition parameters —was investigated in this work. An analysis of characteristics of zones of a Siberian silk moth outbreak under mountain taiga conditions showed that there is a discernable altitudinal belt between 400 and 800 m above sea level where an outbreak develops and trees are damaged. Obviously, this is a necessary but insufficient condition for an outbreak to occur because such altitudinal segments are common in mountain taiga and because not all coniferous forest stands in this altitudinal belt are damaged by the Siberian silk moth. It was demonstrated here that to assess the resistance of forest stands to a pest attack, new parameters can be employed: namely, characteristics of a response of remote sensing variables to changes in LST. By means of these data, an investigator can identify in advance (2–3 years before a pest outbreak) the forest stands that are vulnerable to the pest. Field studies in difficult-to-access taiga forests are not needed to determine these parameters, and therefore, the task of monitoring outbreaks of forest insects is considerably simplified.

The authors tested their proposed method for the early detection of stands vulnerable to insect attacks during a large Siberian silk moth outbreak in the forests of southern Siberia in 2019–2020. It would be useful to test it in areas with other insect outbreaks. Fortunately, Siberian forests do not experience frequent outbreaks of forest insect phylophages that destroy large homogeneous coniferous stands. As outbreaks such as the one studied occur in Siberia every 5–10 years, the approach used in this study can be applied to assess the risk of future outbreaks.

This method uses remote sensing data to assess the stability of plant communities and can also analyze the condition of agricultural crops affected by pests or diseases.

## Figures and Tables

**Table 1:** The proportion of pixels having *U* = 0 on the territory that would become the outbreak zone in the year 2019 and control stands.

Year	Proportion of Pixels with *U* = 0	
	Control Stands	2019 Outbreak Zone
2014	0.52	0.51
2015	0.31	0.42
2016	0.58	0.70
2017	0.45	0.62
2018	0.40	0.64
2019	0.48	0.67
2020	0.24	0.19

## Data Availability

The data presented in this study are available on request from the corresponding author.
